# Study of Corrosion Mechanisms in Corrosive Media and Their Influence on the Absorption Capacity of Fe_2_O_3_/NdFeO_3_ Nanocomposites

**DOI:** 10.3390/nano12132302

**Published:** 2022-07-04

**Authors:** Kayrat K. Kadyrzhanov, Artem L. Kozlovskiy, Kamila Egizbek, Inesh E. Kenzhina, Rauan Sh. Abdinov, Maxim V. Zdorovets

**Affiliations:** 1 Engineering Profile Laboratory, L.N. Gumilyov Eurasian National University, Satpayev St., Nur-Sultan 010008, Kazakhstan; kayrat.kadyrzhanov@mail.ru (K.K.K.); kemelin@mail.ru (K.E.); mzdorovets@gmail.com (M.V.Z.); 2 Laboratory of Solid State Physics, The Institute of Nuclear Physics, Ibragimov St., Almaty 050032, Kazakhstan; kenzhina@physics.kz; 3 ASU Innovations, Kh. Dosmukhamedov Atyray University, Studenchesky Ave., Atyrau 060009, Kazakhstan; r.abdinov@asu.edu.kz; 4 Department of General Physics, Satbayev University, Almaty 050032, Kazakhstan; 5 Department of Intelligent Information Technologies, Ural Federal University, 620075 Yekaterinburg, Russia

**Keywords:** nanocomposites, phase transformations, purification of aqueous media, iron oxide, doping

## Abstract

This paper presents the results of a study of the change in the stability of Fe_2_O_3_/NdFeO_3_ nanocomposites when exposed to aggressive media over a long period of time. The main purpose of these studies is to investigate the mechanisms of degradation and corrosion processes occurring in Fe_2_O_3_/NdFeO_3_ nanocomposites, as well as the influence of the phase composition on the properties and degradation resistance. According to the X-ray phase analysis, it was found that the variation of the initial components leads to the formation of mixed composition nanocomposites with different Fe_2_O_3_/NdFeO_3_ phase ratios. During corrosion tests, it was found that the dominance of the NdFeO_3_ phase in the composition of nanocomposites leads to a decrease in the degradation and amorphization rate of nanostructures by a factor of 1.5–2 compared to structures in which the Fe_2_O_3_ phase dominates. Such a difference in the degradation processes indicates the high stability of two-phase composites. Moreover, in the case of an aqueous medium, nanocomposites dominated by the NdFeO_3_ phase are practically not subjected to corrosion and deterioration of properties. The results obtained helped to determine the resistance of Fe_2_O_3_/NdFeO_3_ nanocomposites to degradation processes caused by exposure to aggressive media, as well as to determine the mechanisms of property changes in the process of degradation. The results of the study of the absorption capacity of Fe_2_O_3_/NdFeO_3_ nanocomposites in the case of the purification of aqueous media from manganese and arsenic showed that a change in the phase ratio in nanocomposites leads to an increase in the absorption efficiency of pollutants from aqueous media.

## 1. Introduction

One of the key parameters for evaluating the application of nanostructures or nanocomposites in practical applications is their resistance to external influences, including corrosion and degradation processes that can lead to negative consequences. The condition of stability of nanostructures entirely determines the time and operating conditions, as well as the scope of their application [1,2,3]. In contrast to massive samples for which, in most cases, the mechanisms of degradation are well enough studied, which has allowed for the development of a number of methods to protect materials from the negative impact of external factors, for nanostructures and nanocomposites, all is not so transparent and clear [4,5]. 

For small nanosized particles, the structure of which may contain microstresses and metastable inclusions associated with the structure formation processes during synthesis, corrosion processes can differ significantly from the processes occurring in bulk samples [6,7]. If in the case of massive samples, most degradation processes in aggressive media are accompanied by the formation of a passivating oxide layer on the material surface, which partially protects the material from further corrosion processes, in nanostructures, the formation of a passivating layer may be difficult for several reasons [8,9,10]. Firstly, due to the small size of the nanostructures, the formed passivation layer may be too small to protect the nanostructures from further corrosion. Secondly, the presence of metastable states in the nanoparticle structure in the form of inclusions can lead to accelerated degradation and oxidation due to external factors causing their accelerated degradation due to their weak stability. Thirdly, the small size and large specific surface area lead to the acceleration of the interaction between the aggressive medium and the nanoparticles surface, which leads to the partial destruction of the surface layer due to the formation of oxide and hydroxide compounds.

In this regard, much attention has been paid in recent years to studies aimed at a comprehensive study of the resistance of nanostructures to degradation mechanisms and corrosion processes occurring in nanomaterials [11,12]. A number of studies have shown that the degradation of nanostructures in conditions of aggressive media proceeds according to the mechanisms of pitting corrosion with the formation of ulcerous inclusions or growths leading to partial destruction of nanostructures [13,14,15]. At the same time, as it was shown in the works, the corrosion mechanisms depend very strongly on the phase composition of the initial structures, as well as on the degree of structural ordering [16,17]. Metal oxide nanoparticles based on ferrite or orthoferrite compounds are the most unstable to corrosion processes and degradation mechanisms. In turn, these oxide nanostructures based on iron, nickel or cobalt are of great interest from a practical point of view due to their great potential for application in photocatalysis, biomedicine, absorption and purification of aqueous media, accumulator batteries, etc. [18,19,20]. The interest in these nanostructures is primarily due to their magnetic and structural properties, which allow them to be used in such a wide range of practical applications. It should be noted that most of these applications are accompanied by operation in conditions of aggressive environments or external influences, which requires a comprehensive study of corrosion and degradation mechanisms due to the specifics of nanostructures [21,22,23].

One way to increase the corrosion resistance of iron-based metal oxide nanoparticles is to create composite nanostructures by doping them with more stable magnetic elements, which will not only increase the degradation resistance, but also preserve the magnetic properties [24,25]. 

The main purpose of this work is to study the mechanisms of corrosion and degradation of Fe_2_O_3_/NdFeO_3_ nanocomposites under corrosive media, as well as to determine the absorption properties when manganese and arsenic are absorbed. The choice of Fe_2_O_3_/NdFeO_3_ nanocomposites as samples for the study is due to their wide practical application in the field of photocatalysts and bases for the aqueous media purification from heavy metals. Interest in these structures is due to the possibility of their removal from aqueous media by trapping with magnets, thereby excluding the possibility of contamination of water sources with photocatalyst products or their residues. Additionally, iron-containing nanostructures are quite harmless and do not cause mutational consequences when in contact with living organisms, which eliminates negative consequences in case of prolonged exposure to aquatic environments. However, long-term use requires a clear understanding of the resistance of nanostructures to processes that occur when they interact with aqueous or aggressive media, which are accompanied by corrosion and degradation processes. It is also worth noting that, unlike massive samples, corrosion mechanisms for nanostructures can proceed much faster due to their small size and structural features of nanomaterials. An analysis of the literature data showed [26,27,28] that there are quite a few works devoted to a detailed study of corrosion processes in iron-containing nanostructures, which opens wide opportunities for studying this problem. In addition, the relevance of this study consists in the assessment of the NdFeO_3_ phase formation effect on the corrosion and degradation resistance mechanisms, as well as the catalytic activity of iron-containing nanocomposites.

## 2. Experimental Part

Nanostructures based on iron oxide (Fe_2_O_3_) with hematite structure, mixed with Nd_2_O_3_ with different concentration to obtain composite nanostructures, were selected as objects of study. The synthesis of nanocomposites was performed in two consecutive stages, including chemical precipitation of Fe_3_O_4_ nanoparticles, according to the method described in [29,30] and subsequent mechanochemical synthesis consisting in mixing Fe_3_O_4_ and Nd_2_O_3_ nanoparticles in specified proportions, followed by grinding and thermal annealing. The concentration of the sample components was Fe_3_O_4_:Nd_2_O_3_ → 0.9:0.1; 0.8:0.2; 0.7:0.3; 0.6:0.4 and 0.5:0.5 in mass ratio. For further convenience, the samples were numbered as follows: A1 → 0.9:0.1; A2 → 0.8:0.2; A3 → 0.7:0.3; A4 → 0.6:0.4; A5 → 0.5:0.5. Grinding was carried out in a planetary mill PULVERISETTE 6 (Fritsch international, Idar-Oberstein, Germany) at a grinding speed of 400 rpm for 1 h. After grinding, the samples were annealed at 800 °C for 8 h followed by cooling of the furnace and the samples in it for 24 h in order to avoid hardening processes that can occur due to rapid temperature changes in the case of sharp withdrawal of samples. The choice of the temperature regime is due to the initialization of the phase transformations of the type Fe_3_O_4_ → Fe_2_O_3_, accompanied by processes of structural ordering at high temperatures [30]. The choice of component concentrations for the synthesis is due to the possibility of obtaining complex oxides such as NdFeO_3_ or substitution solid solutions.

The phase composition of the synthesized nanocomposites under study was studied using the X-ray phase analysis method implemented on a D8 Advance ECO X-ray diffractometer (Bruker, Karlsruhe, Germany). The Diffrac EVA v.4.2 software (Bruker, Karlsruhe, Germany) was used to determine the structural parameters as well as the phases and their contributions. The PDF-2 database (2016) was used to determine the phase composition. The phases were established by selecting the maximum correspondence between the position of the diffraction peaks and the card value data from PDF-2, considering a priori information about the structure and the method for obtaining it. To determine the phase content, the Rietveld method was used and the calculation Formula (1):

(1) $$V_{\mathrm{admixture}}=\frac{RI_{\mathrm{phase}}}{I_{\mathrm{admixture}}+RI_{phase}},$$

*I_phase_* is the average integrated intensity of the main phase of the diffraction line, *I_admixture_* is the average integrated intensity of the additional phase and *R* is the structural coefficient equal to 1.45.

Refinement of the crystal lattice parameters was carried out using the Nelson–Taylor equation.

Determination of the phase composition was carried out by evaluating the contributions of diffraction reflections for the established phases, with further determination of their weight value and determination of the ratio.

The morphological features of the synthesized nanocomposites were studied using scanning electron microscopy realized with a Hitachi TM 3030 microscope (Hitachi, Japan).

Particle size determination was carried out using an Analysette 22 MicroTec plus laser diffraction particle size analyzer (FRITSCH NanoTec, Karlsruhe, Germany).

The corrosion resistance tests of Fe_2_O_3_/NdFeO_3_ nanocomposites under corrosive media were carried out over a time interval of 10 days. Solutions of 1M NaOH and 0.1 M HCl, and water chosen as the comparison medium, were chosen as the test media. The choice of the model media was caused by the possibilities of simulation of the corrosion processes under the conditions closest to the real operating conditions of nanocomposites in various practical applications.

Evaluation of the efficiency of the purification of aqueous media from heavy metals, in particular, manganese and arsenic, was conducted by performing model experiments aimed at determining changes in the optical density of aqueous solutions, with manganese and arsenic dissolved in them at given concentrations. Determination of changes in the optical density of solutions was carried out using the device “Fluorat-02” (Lumex, Moscow, Russia). Synthesized nanocomposites weighing 0.01 g placed in the model solution and subjected to constant stirring for 1 h were used as catalysts. After this time, aliquots were taken to measure optical density and determine the concentration of substances in the solution. 

## 3. Results and Discussion

**Figure 1:**
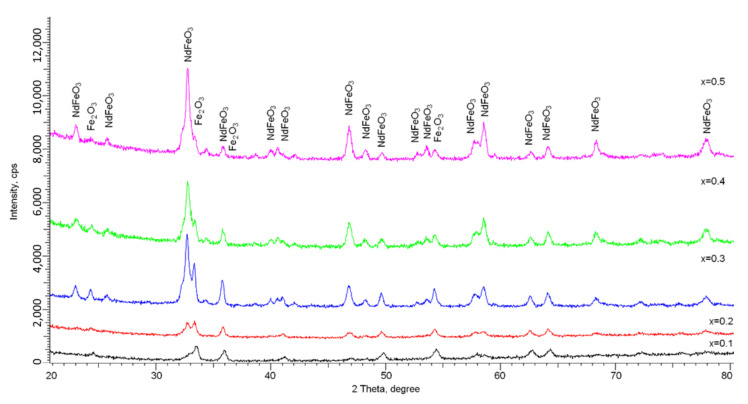
X-ray diffractograms of Fe_2_O_3_/NdFeO_3_ nanocomposites depending on the components ratio.

Figure 1 shows the X-ray diffraction results of Fe_2_O_3_/NdFeO_3_ nanocomposites depending on the concentration of the components used in the synthesis process.

The analysis of the obtained diffractogram data revealed that all the observed diffraction reflections are characteristic of the Fe_2_O_3_ phase with a hematite structure and hexagonal lattice type and the NdFeO_3_ phase with an orthorhombic lattice type. According to the presented data of the X-ray diffraction, it is established that the main changes observed on the diffraction patterns are related to the changes in the contributions of the two phases Fe_2_O_3_ and NdFeO_3_ depending on the content of the components in the synthesized samples. Variation of the component ratio in the initial mixtures during the preparation of nanocomposites leads to a change in the ratio of the Fe_2_O_3_/NdFeO_3_ phases. It has been established that the Fe_2_O_3_ phase is the main phase in the structure of samples with a component ratio of 0.9:0.1, while at a component ratio of 0.5:0.5, the NdFeO_3_ phase dominates. At the same time, a change in the component ratio does not lead to the appearance of diffraction reflections characteristic of other phases, which indicates that the selected synthesis conditions do not lead to the formation of other phases than the established ones.

**Figure 2:**
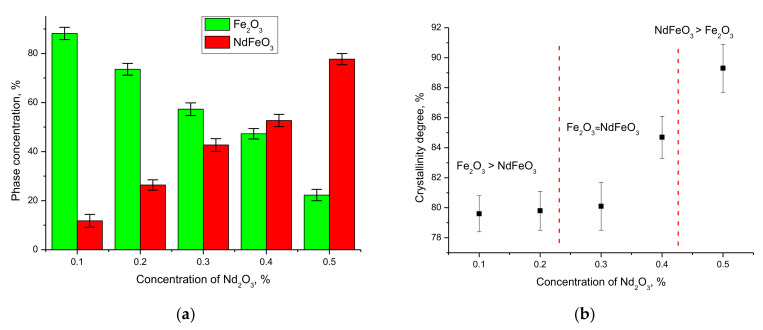
(**a**) Phase diagram of Fe_2_O_3_/NdFeO_3_ nanocomposites. (**b**) Graph of the change in the degree of crystallinity of Fe_2_O_3_/NdFeO_3_ nanocomposites depending on the components content.

The analysis of the obtained X-ray diffractograms showed that at Nd_2_O_3_ concentrations in the initial mixtures equal to 0.1 and 0.2, the Fe_2_O_3_ phase with a hematite structure is the dominant phase in the structure. At the same time, the presence of peaks characteristic of the NdFeO_3_ phase indicates that the mechanochemical synthesis and subsequent thermal annealing lead to partial replacement of iron ions by neodymium ions in the crystal lattice, and to the subsequent formation of a substitutional solid solution phase of the NdFeO_3_ type. The increase in the Nd_2_O_3_ concentration to 0.3 and higher leads first to a phase ratio close to that of 1:1, and at an Nd_2_O_3_ concentration equal to 0.5 to the dominance of the NdFeO_3_ phase, which indicates a high degree of substitution by neodymium ions of iron ions in the crystal lattice nodes (see Figure 2a). The presence of the Fe_2_O_3_ phase even at high concentrations of Nd_2_O_3_ indicates that the processes of phase transformations with the formation of the substitution phase do not proceed to the end. It is also worth noting that even at high concentrations of Nd_2_O_3_ in the initial mixtures, no peaks characteristic of this phase were observed, indicating complete dissolution under these synthesis conditions.

As can be seen from the presented data in Figure 2b, a change in the Fe_2_O_3_/NdFeO_3_ phase ratio followed by the NdFeO_3_ phase dominance in the structure leads to an increase in the degree of structural ordering (degree of crystallinity), which indicates that the phase composition change has an effect on the structure ordering of nanocomposites. At the same time, the dominance of the NdFeO_3_ phase leads to an ordering of the structure by 10–12%, and the analysis of the diffraction line shapes directly indicates a decrease in the deformation contributions to the structure when the phase composition is changed. This behavior of structural parameters can further have a significant impact on the resistance of nanocomposites to corrosive environments.

Table 1 presents the results of estimating the structural parameters of the crystal lattice (refinement of the crystal lattice parameters was carried out using the DiffracEVA v.4.2 program code).

According to the data presented in Table 1, an increase in the Nd_2_O_3_ concentration in the nanocomposites leads to an increase in the crystal lattice parameters for the Fe_2_O_3_ phase, which may be due to the processes of replacement of iron ions by neodymium ions. Additionally, differences in the crystal lattice parameters can be due to differences in the ionic radii of Nd^3+^ and Fe^3+^. For the NdFeO_3_ phase, the increase in its content in the structure is observed to decrease the crystal lattice parameters and its volume, which indicates an ordering of the structure and reduction of amorphous-like or disordered regions in the structure of nanocomposites.

**Figure 3:**
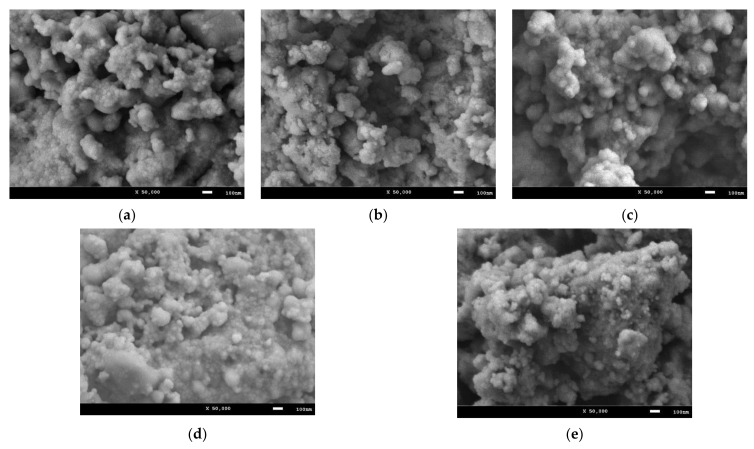
SEM images of the studied Fe_2_O_3_/NdFeO_3_ nanocomposites: (**a**) A1; (**b**) A2; (**c**) A3; (**d**) A4; (**e**) A5.

Figure 3 shows the results of the study of morphological features of the synthesized nanocomposites, reflecting the dynamics of changes in the size and shape of the grains of which the composites are composed depending on the ratio of the components used. As can be seen from the data presented, the morphology of the composites obtained are dendrite-like structures consisting of sphere-like grains with sizes ranging from 30 to 70 nm. At the same time, changes in the ratio of the components in the composition lead to an increase in the packing density of the synthesized nanocomposites, with the formation of large inclusions consisting of smaller grains. It should also be noted that in the case of the concentration of 0.4–0.5, in the structure of the obtained nanocomposites, the presence of a small number of grains with sizes no larger than 20 nm is observed, which, when comparing the X-ray diffraction data in assessing the crystallite sizes, is close to the values obtained for the Fe_2_O_3_ phase.

**Figure 4:**
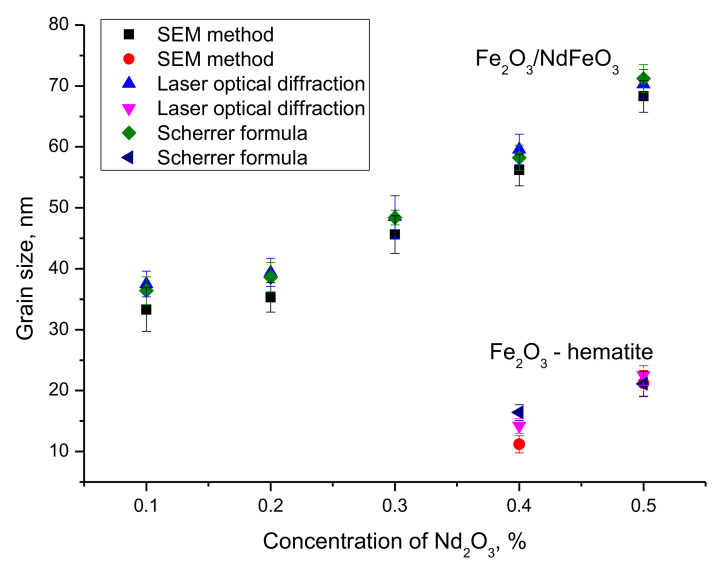
Results of grain size measurements by different methods.

Figure 4 shows the results of the comparative analysis of grain (crystallite) sizes obtained by scanning electron microscopy, X-ray diffraction and laser optical diffraction. Size estimation using scanning electron microscopy was carried out by processing the obtained images with direct grain size calculation and construction of size diagrams in order to determine the mean value and standard deviation. Determination of the grain sizes using the laser optical diffraction method was carried out according to the standard technique of dispersion of the obtained nanocomposites in an aqueous solution and subsequent imaging and construction of dimensional diagrams. Grain (crystallite) size determination by X-ray diffraction was carried out using the method proposed by Scherrer. 

As can be seen from the data presented in Figure 4, the estimation of grain sizes by different methods has a very good agreement, with all the results of deviations of changes by different methods being within the error, which indicates a high accuracy of grain size determination. The general trend of grain size changes indicates that the change in the phase composition of nanoparticles is accompanied by a slight increase in size for concentrations of 0.1–0.3, and a significant increase in grain size at Nd_2_O_3_ concentrations in the composition equal to 0.4–0.5. For these concentrations, according to all obtained data from all methods, there is a separation of sizes into two characteristic ranges of 60–70 nm and 10–20 nm, whose contribution is estimated as 10–15% of the total number of measured particles (according to SEM images and laser optical diffraction results). According to XRD data, these sizes correspond to the peaks characteristic of the Fe_2_O_3_ phase. Thus, it can be assumed that in the case of dominance of the NdFeO_3_ phase, the Fe_2_O_3_ phase can decrease with the formation of small particles, which, according to the data of scanning electron microscopy, are in the form of small growths around larger particles. In the case of Nd_2_O_3_ concentrations equal to 0.1–0.3, size difference is not observed, which indicates that the obtained composites have the same size and represent two-phase particles.

One of the ways to assess the mechanisms of nanostructures degradation is the method of measuring changes in the mass of the samples under study during the time spent in the medium. These changes characterize the oxidation processes, including the formation of oxide films or growths on the surface of nanostructures, as well as degradation mechanisms accompanied by partial destruction of the samples during corrosion. Measurement of the mass of the samples before and after the corrosion resistance tests was carried out using high-precision laboratory analytical scales (accuracy class 0.0001 g) in five parallels in order to determine the error of measurements.

**Figure 5:**
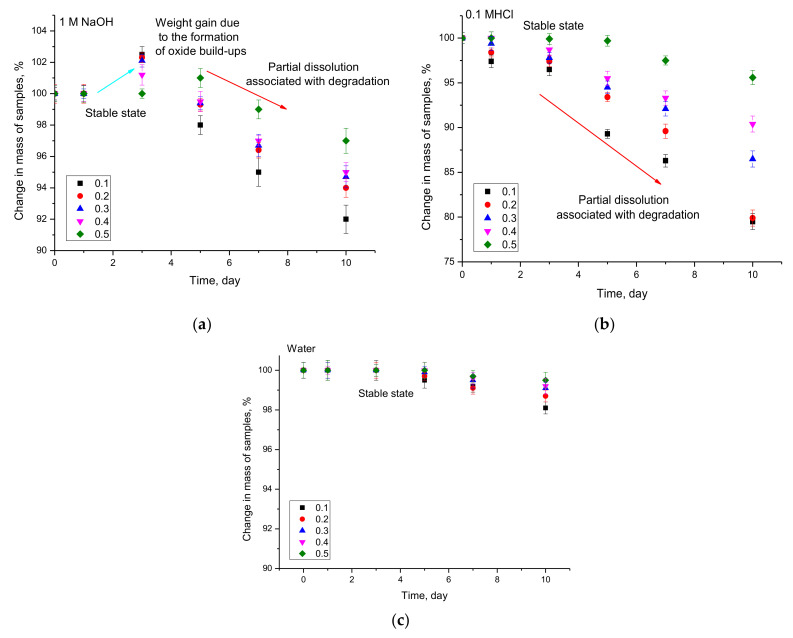
Results of mass changes of samples during corrosion tests in different media: (**a**) 1M NaOH; (**b**) 0.1 M HCl; (**c**) water.

Figure 5 shows the results of changes in the mass of the investigated samples during the corrosion tests in different environments.

**Figure 6:**
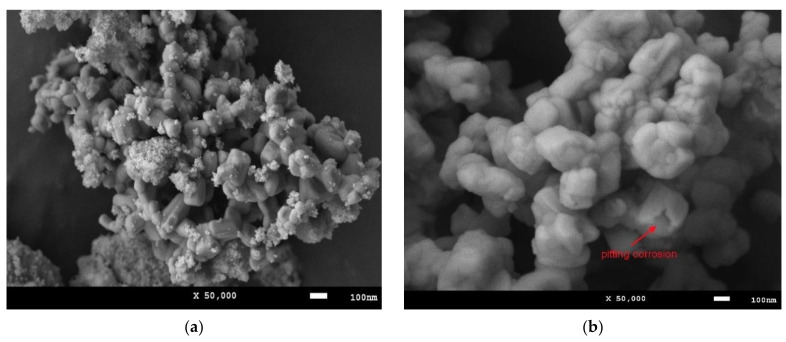
(**a**) Example of oxide growths formation on Fe_2_O_3_/NdFeO_3_ nanocomposites surface (Nd_2_O_3_ = 0.1) as a result of corrosion processes in 1 M NaOH medium. (**b**) Example of pitting corrosion in Fe_2_O_3_/NdFeO_3_ nanocomposites (Nd_2_O_3_ = 0.1) after 10 days in 0.1 M HCl medium.

In the case of the model medium 1M NaOH, characterized by an alkaline environment, the mass change of the samples occurs in three stages. The first stage is characterized by the absence of mass changes, which indicates the stability of nanocomposites to the processes of corrosion and degradation in interaction with the medium. The second stage is characterized by a slight increase in sample mass (no more than 1–2%), which is caused by the processes of formation of growths on the surface of nanocomposites as a result of their interaction with the medium. Such behavior of sample mass changes is characteristic as in the case of massive samples by formation of oxide passive layers, but due to the small size of nanocomposites, these films or layers cannot form a single structure but are formed as small feather-like growths (data in Figure 6). The third stage of changes is characterized by a decrease in sample mass, which indicates that corrosion processes proceed by partial destruction of nanocomposites associated with the formation of pittings or ulcerated inclusions, accompanied by amorphization and destruction of the crystal structure. According to the X-ray phase analysis, the reduction of the degree of structural ordering of the studied samples after 10 days in a 1M NaOH medium is more than 10–20% depending on the phase composition of the samples, indicating their destruction and amorphization during the interaction with the medium. Herewith, the increase in the contribution of the NdFeO_3_ phase in the composition of nanoparticles leads to an increase in the resistance to structural degradation characterized by smaller changes in the mass of the samples during all the tests. Amorphization was confirmed by the results of the X-ray phase analysis, according to which, after the samples are exposed to aggressive media, there is an increase in the halo contribution characteristic of amorphous inclusions, as well as a strong deformation of diffraction reflections.

In the case of the acid medium 0.1 M HCl, the dynamics of sample mass changes is characterized by two stages: a stable absence of sample mass changes and its decrease characteristic of degradation and partial destruction of nanocomposites. The comparative analysis of mass changes for 0.1 M HCl and 1M NaOH media shows that nanocomposites are more stable in alkaline media than in acidic ones, which is confirmed by mass changes.

In the case of the aqueous medium, there is almost no change in mass, indicating that the synthesized nanocomposites are resistant to ordinary water, and corrosion processes in it are too slow and weakly manifested only after 7 days of testing, while the stable mass preservation of samples for the alkaline and acid medium was no more than 1–3 days, depending on the phase composition of nanoparticles.

Figure 6 shows SEM images, given as an example, demonstrating morphological changes as a result of the corrosion processes. In the case of the model medium 1M NaOH, the increase in mass of the samples is associated with the formation of oxide or hydroxide outgrowths, the presence of which was confirmed by the X-ray phase analysis data. The content of impurity phases in the samples does not exceed 2–3% and are identified on the diffractograms in the form of low-intensity reflections in the area of small angles. In the case of the 0.1 M HCl medium after 10 days, it was found that pores or microsculptures characteristic of pitting corrosion processes are observed in the structure of nanocomposites.

The degree of change in sample mass (*DMC*) after 10 days of corrosion was estimated using Formula (2):

(2) $$DMC=\frac{m_{0}-m_{10}}{m_{0}}\times 100\%$$

where *m*_0_ and *m*_10_ are the mass values of the samples in the initial state and after being in the medium for 10 days.

The efficiency of corrosion resistance (*CRP*) was evaluated using Formula (3) based on the change in the ratio of mass changes of the samples after 10 days of corrosion.

(3) $$CRP=\frac{DMC_{x}-DMC_{0.1}}{DMC_{0.1}}\times 100\%$$

**Figure 7:**
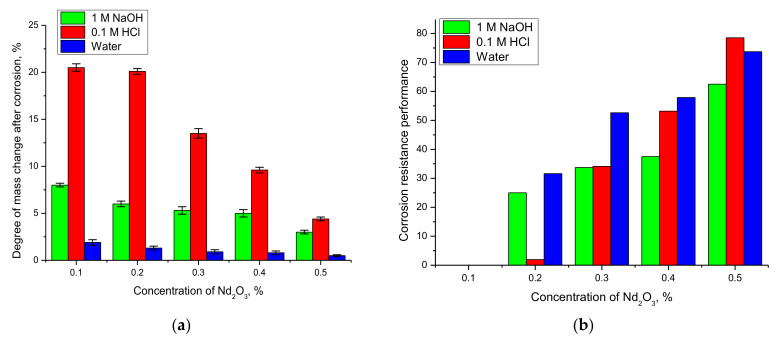
(**a**) Results of sample mass change during corrosion after 10 days. (**b**) Results of corrosion resistance evaluation depending on the phase composition of nanoparticles.

*DMC*_0.1_, *DMC_x_*—results of mass changes of samples with different components content. The results of calculations using Formulas (1) and (2) are shown in Figure 7.

The general trends of mass changes and the evaluation of the corrosion resistance efficiency show a strong dependence of the corrosion rate and degradation of nanocomposites on the phase composition and phase dominance in the nanocomposite composition. In the case of nanocomposites dominated by the Fe_2_O_3_ phase composition, the corrosion processes are much faster than in the case where the NdFeO_3_ phase dominates the structure. This behavior can be explained by the fact that Fe_2_O_3_ nanoparticles have a rather low resistance to oxidation processes and are able to degrade when exposed to acids and alkalis, forming oxide or hydroxide compounds, which, due to their instability, lead to the destruction of nanoparticles (data in Figure 6b). Additionally important in the stability of nanocomposites is the degree of structural ordering (degree of crystallinity) in the initial state, which characterizes the presence of metastable or disordered inclusions in the structure. In the case of Nd_2_O_3_ concentration = 0.1–0.2 in the nanocomposites, the degree of crystallinity, according to the X-ray phase analysis data, is much lower than for the structures in which the NdFeO_3_ phase dominates (see data in Figure 2b). The presence of a large number of disordered inclusions in the initial state of nanocomposites can lead to acceleration of degradation and oxidation processes, which was observed in the case of sample mass changes. The presence of these disordered inclusions was established during diffraction reflection shape analysis, during which it was determined that the shape of diffraction reflections is strongly distorted and asymmetric, which indicates a strong deformation of the structure. At the same time, the dominance of the NdFeO_3_ phase in the nanocomposite structure leading to an increase in the degree of structural ordering leads to a decrease in the rate of corrosion and degradation of the material, which is reflected in a decrease in sample mass change as well as an increase in resistance to corrosion (data in Figure 7b).

One of the most promising applications of magnetic nanocomposites is the purification of aqueous media from heavy metals by absorption and their further extraction for recycling or decomposition. In most cases of application of catalysts, efficiency is estimated by the change in data of optical density of aqueous media or model solutions in which pollutants are dissolved, as well as stability and preservation of purification efficiency during long test cycles. In this case, the absorption efficiency in most cases of known catalysts does not exceed 50–70%, and is characterized by a low degree of resistance to degradation of nanocatalysts, which does not contribute to an increase of their operating time. 

**Figure 8:**
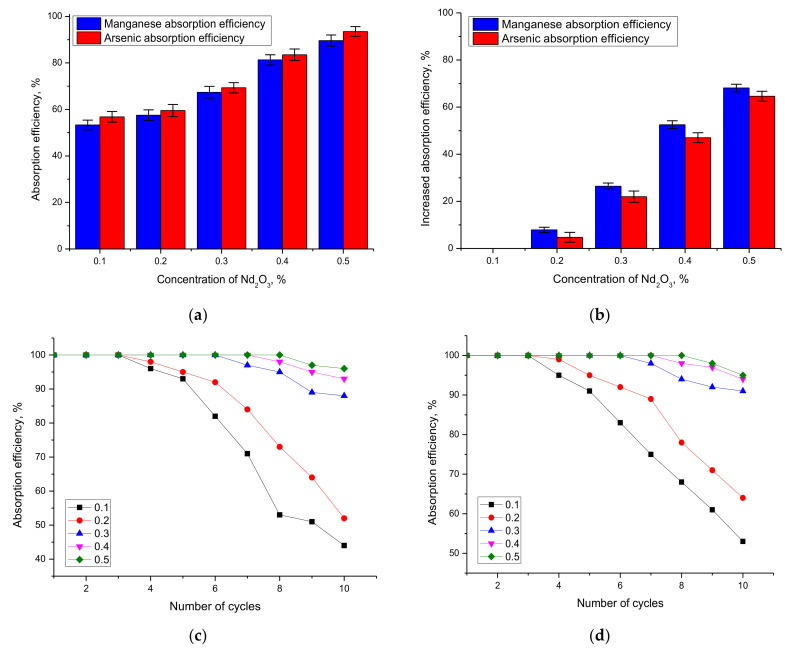
(**a**) Absorption efficiency results for manganese and arsenic. (**b**) Absorption efficiency results as a function of phase composition. (**c**) Absorption efficiency dynamics as a function of number of consecutive cycles for manganese purification. (**d**) Absorption efficiency dynamics as a function of number of consecutive cycles for arsenic purification.

Figure 8 shows the results of assessing the absorption efficiency of manganese and arsenic by the studied nanocomposites. The absorption efficiency of heavy metals was estimated using the Formula (4):

(4) $$Absorption\_effeciency=\left(\frac{C_{0}-C}{C_{0}}\right)*100\%$$

where *C*_0_ and *C*—concentrations of manganese and arsenic before and after absorption.

As can be seen from the data presented, when the structure of Fe_2_O_3_/NdFeO_3_ nanocomposites is dominated by the Fe_2_O_3_ phase, the absorption efficiency of manganese and arsenic does not exceed 57–60%, which is a very intermediate result and does not characterize these structures as highly effective catalysts for cleaning aqueous media. However, when in the structure of Fe_2_O_3_/NdFeO_3_ nanocomposites, the NdFeO_3_ phase dominates and the absorption efficiency increases to 80–90%. In this case, according to the data presented in Figure 8b, the absorption efficiency of manganese and arsenic in the case of comparison with samples in which the dominant phase Fe_2_O_3_ is more than 60% for samples with the dominant phase NdFeO_3_. It is also worth noting that for both model solutions, the absorption efficiency is about the same, with a slight increase for arsenic absorption. This indicates that Fe_2_O_3_/NdFeO_3_ nanocomposites have comparable efficiency in the purification of aqueous media from both manganese and arsenic, which opens up the possibility of their use as catalysts in the future.

Analysis of the evaluation of repeatability and preservation of absorption efficiency results during repeated cycling of Fe_2_O_3_/NdFeO_3_ nanocomposites for aqueous media treatment presented in Figure 8c–d showed that synthesized catalysts dominated by the NdFeO_3_ phase fully preserve their absorption efficiency during 7–8 cycles with a slight decrease (no more than 5–7%) after 8 cycles, which indicates high stability of aqueous media treatment during long-term operation.

## 4. Conclusions

In conclusion of the research, we can make the following conclusions and summarize the results.

1. The proposed method of synthesis makes it possible to obtain Fe_2_O_3_/NdFeO_3_ nanocomposites with different variation of the phase ratio as well as structural parameters. Moreover, an increase in the NdFeO_3_ contribution leads to an increase in the degree of structural ordering as well as an insignificant change in the grain size.
2. During experiments on corrosion resistance, it was found that the dominance of the NdFeO_3_ phase in the structure of nanocomposites leads to an increase in destruction resistance and a decrease in corrosion rate by 1.5–2 times. At the same time, in the case of aggressive media, the initial stages of corrosion processes are accompanied by the formation of oxide and hydroxide growths (the content of which is no more than 3–5%), which have an amorphous structure.
3. As a result of tests on aqueous media purification from manganese and arsenic, the dependence of the effect of the Fe_2_O_3_/NdFeO_3_ phase ratio in nanocomposites on the absorbent capacity and aqueous media purification degree was established. It has been determined that an increase in the content of the NdFeO_3_ phase in nanocomposites leads to an increase in the aqueous media purification efficiency by 30–35%. At the same time, for composites with the dominant NdFeO_3_ phase, the absorption efficiency is maintained for 7–8 successive cycles.

Further research perspectives will be aimed at studying the influence of the phase composition on the magnetic properties of nanocomposites, as well as expanding the range of their practical applications in photocatalysis and purification of aqueous media.

## Figures and Tables

**Table 1:** Data of crystal lattice parameters.

Phase	Lattice Parameter				
	Concentration of Nd_2_O_3_ in Nanocomposites				
	0.1	0.2	0.3	0.4	0.5
	A1	A2	A3	A4	A5
Fe_2_O_3_, Hexagonal, R-3c(167)	a = 4.9843 ± 0.0014 Å, c = 13.6853 ± 0.0016 Å, V = 294.43 Å^3^	a = 4.9922 ± 0.0024 Å, b = 13.7017 ± 0.0015 Å, V = 295.73 Å^3^	a = 4.9971 ± 0.0021 Å, c = 13.7259 ± 0.0015 Å, V = 296.83 Å^3^	a = 5.0118 ± 0.0017 Å, c = 13.7744 ± 0.0021 Å, V = 299.22 Å^3^	a = 5.0285 ± 0.0022 Å, c = 13.7744 ± 0.0015 Å, V = 301.63 Å^3^
NdFeO_3_, Orthorhombic, Pnma(62)	a = 5.5547 ± 0.0015 Å, b = 7.7391 ± 0.0014 Å, c = 5.4329 ± 0.0021 Å, V = 233.55 Å^3^	a = 5.5459 ± 0.0016 Å, b = 7.7313 ± 0.0022 Å, c = 5.4168 ± 0.0027 Å, V = 232.25 Å^3^	a = 5.5317 ± 0.0021 Å, b = 7.7089 ± 0.0024 Å, c = 5.4051 ± 0.0022Å, V = 230.48 Å^3^	a = 5.5198 ± 0.0022 Å, b = 7.6889 ± 0.0017 Å, c = 5.3934 ± 0.0024 Å, V = 228.90 Å^3^	a = 5.5382 ± 0.0013 Å, b = 7.6745 ± 0.0012 Å, c = 5.3830 ± 0.0016 Å, V = 226.48 Å^3^
	**Phase concentration, weight%**				
Fe_2_O_3_, Hexagonal, R-3c(167)	88.2 ± 2.5	73.6 ± 2.4	57.3 ± 2.4	47.3 ± 2.1	22.3 ± 2.3
NdFeO_3_, Orthorhombic, Pnma(62)	11.8 ± 2.6	26.4 ± 2.1	42.7 ± 2.6	52.7 ± 2.5	77.7 ± 2.3

## Data Availability

Not applicable.
